# 加速溶剂萃取-分子筛固相萃取-气相色谱-串联质谱法测定土壤中多氯萘

**DOI:** 10.3724/SP.J.1123.2021.12030

**Published:** 2022-10-08

**Authors:** Jing JIN, Hongyuan LIU, Huifu XUE, Jing YANG, Chunhua QU, Huilian MA, Jiping CHEN

**Affiliations:** 1.中国科学院大连化学物理研究所, 中国科学院分离分析化学重点实验室, 辽宁 大连 116023; 1. CAS Key Laboratory of Separation Science for Analytical Chemistry, Dalian Institute of Chemical Physics, Chinese Academy of Sciences, Dalian 116023, China; 2.沈阳药科大学, 辽宁 沈阳 110000; 2. Shenyang Pharmaceutical University, Shenyang 110000, China; 3.新兴能源科技有限公司, 辽宁 大连 116023; 3. SYN Energy Technology Co., Ltd., Dalian 116023, China; 4.中国环境监测总站, 北京 100012; 4. China National Environmental Monitoring Centre, Beijing 100012, China; 5.重庆中医药学院, 重庆 402760; 5. Chongqing College of Traditional Chinese Medicine, Chongqing 402760, China

**Keywords:** 固相萃取, 气相色谱-串联质谱, 多氯萘, 分子筛, 土壤, solid-phase extraction (SPE), gas chromatography-tandem mass spectrometry (GC-MS/MS), polychlorinated naphthalenes (PCNs), molecular sieves, soil

## Abstract

新污染物引发的环境和健康风险正逐步受到社会各界的广泛关注,我国第十四个五年规划和2035年远景目标纲要明确“重视新污染物治理”。作为新型的持久性有机污染物,多氯萘(PCNs)在土壤中通常处于痕量水平,一般需要经过多层硅胶柱/氧化铝柱等复杂的净化方法,再结合有效的分析手段才能实现准确测定。关注土壤中多氯萘分离分析方法可以为掌握和监管其在土壤中的污染状况提供技术和方法支持。研究以13X分子筛作为固相萃取吸附剂,评价了其对多氯萘的净化效果。研究发现:使用正己烷作为上样溶剂和淋洗剂,10 mL二氯甲烷/正己烷(2∶15, v/v)为洗脱溶剂,可以实现PCNs与脂类大分子等干扰物的选择性分离,且多氯萘内标的平均回收率为56.1%~88.0%。与凝胶渗透色谱法、弗罗里硅土固相萃取柱以及多层硅胶柱/氧化铝柱相比,13X分子筛对土壤提取液的净化效果优于前两种净化方法,可以获得与多层硅胶/氧化铝柱相近的净化效果(53.0%~117.0%),而且操作更加简单,环境更加友好,分析成本大幅度下降。在此基础之上,建立了加速溶剂萃取-分子筛固相萃取,结合气相色谱-三重四极杆质谱法测定土壤中PCNs的分析方法。PCNs同族体的方法检出限为0.009~0.6 ng/g。采用基质加标法评价了本方法的精密度和准确度,CN-3、13、42、46、52、53、73、75在低、中、高加标水平下的平均加标回收率分别为70%~128%、71%~115%和61%~114%,测定结果的相对标准偏差分别为4.2%~23%、6.5%~31%和4.7%~22%,满足痕量分析的要求且平行性较好。从整个分析流程来看,13X分子筛有望成为新污染物净化的新型固相萃取吸附剂,并在土壤新污染物普查中发挥重要作用。

## Abstract (en)

Emerging pollutants (EPs) are chemical substances that are commonly not regulated and can be detected at low or very low concentrations. However, EPs have triggered special concern because their long-term adverse effects on the environment and human health remain unknown. Most EPs show biological toxicity, environmental persistence, and bioaccumulation. Even at low concentrations in the environment, EPs may pose significant environmental and health risks. Therefore, their treatment has been explicitly included in the 14th Five Year Plan for National Economic and Social Development of the People’s Republic of China and the Outline of the Long-term Goals for 2035. Soil is a source of pollutants, and its quality is directly related to economic development, ecological security, and people’s livelihood. At present, China’s soil environmental monitoring system is not perfect, and the ability to monitor these new organic pollutants is lagging. Therefore, to strengthen the supervision of construction and agricultural land soil environments, it is essential to strengthen the soil environment monitoring ability for these EPs and establish a reliable, steady, and economic analysis method, including their separation and analysis methods in soil. Polychlorinated naphthalenes (PCNs) have received considerable attention as emerging halogenated compounds. They were listed in Annexes A and C of the Stockholm Convention on persistent organic pollutants (POPs) in 2015 because of their persistence, multimedia fate, and toxicity. PCNs have now been detected in the surrounding soils. Owing to their trace levels in complex soil, high requirements have been put forward for the pretreatment and instrument analysis of PCNs.

This study aims to develop a new method for the selective purification of PCNs in soil, which can not only effectively remove lipids and other interferences in soil but also effectively reduce time, labor, and material costs in the pre-treatment process. Based on the physicochemical properties of the 13X molecular sieve, it was explored to purify soil-extracts as solid-phase extraction (SPE) sorbents. With *n*-hexane as the loading and rinsing solvent, 10 mL of a dichloromethane/*n*-hexane mixture (2∶15, v/v) was used to elute the PCNs. Moreover, selective separation of target substances from lipid macromolecules and other interferences could be achieved simultaneously. For the selective separation of PCNs, the average recovery of the internal standard could reach 56.1% to 88.0%. 13X molecular sieves are superior to gel permeation chromatography (GPC) and Florisil SPE, and they exhibit good cleanup efficiency similar to a multilayer silica gel/alumina column (53.0%-117.0%). Although the obtained recoveries are not as high as those obtained with a multilayer silica gel/alumina column, 13X molecular sieves have advantages in terms of simple operation, environmental friendliness, and low cost. Based on these fundamental experiments, accelerated solvent extraction was used to extract targets in soil, molecular sieves were used as SPE sorbents for purification, and GC-MS/MS was employed for PCN analysis. This method was developed as a systematic analytical method for PCNs determination. The method detection limits (MDLs) for PCN homologs were in the range of 0.009-0.6 ng/g. The precision and accuracy of the method were evaluated using spiked matrices. At three spiked levels (4, 10, and 18 ng), the recoveries of PCNs (CN-3, 13, 42, 46, 52, 53, 73, and 75) were 70%-128%, 71%-115%, and 61%-114%, respectively, and the corresponding relative standard derivations were 4.2%-23%, 6.5%-31% and 4.7%-22%. Thus, this method meets the requirements of trace analysis and shows acceptable parallelism, sensitivity, accuracy, and precision, thus being feasible for the analysis of emerging pollutant. The method is expected to play an important role in sample pretreatment in the future, especially for the nationwide investigation of soil pollution.

土壤作为各类污染物的汇集地,其质量状况直接关系到经济发展、生态安全和民生福祉。近年来,我国土壤污染防治工作取得积极进展,环境质量持续改善。但与此同时,新污染物引发的环境和健康风险正逐步受到社会各界的广泛关注,“重视新污染物治理”已明确写入《中华人民共和国国民经济和社会发展第十四个五年规划和2035年远景目标纲要》。新污染物不同于常规污染物,指新近发现或被关注,对生态环境或人体健康存在风险,尚未纳入管理或者现有管理措施不足以有效防控其风险的污染物。它们的主要来源是有毒有害化学物质的生产和使用。多氯萘(polychlorinated naphthalenes, PCNs)具有与2,3,7,8-四氯二苯并对二噁英等化合物相似的化学结构,是工业热过程的主要污染物之一,作为一种新污染物已受到广泛关注。目前,我国土壤环境监测能力尚不能及时掌控全国和区域土壤环境状况,土壤环境监测体系尚不完善,对于这些新污染物的监测能力更是相对滞后。因此,为加强建设和农业用地土壤环境监管,管控污染地块对人体健康的风险,保障人居环境安全,亟待加强这些新污染物的土壤环境监测能力,建立可靠、稳健、经济的分析方法,从而有助于推动新污染物监测体系的完善,为完成国际履约提供技术和方法支持。

PCNs在土壤中的含量范围通常为pg/g~ng/g,虽然污染场地土壤中其含量相对较高(μg/g),但总体上仍然处于痕量水平^[1⇓⇓⇓⇓⇓⇓⇓⇓⇓-11]^。而土壤介质比较复杂,一般需通过净化才能实现其灵敏、准确检测。一般而言,土壤提取液通常采用层析柱、凝胶渗透色谱或固相萃取柱等方式达到净化目的^[12⇓⇓⇓⇓⇓⇓-19]^。层析柱法常用吸附剂材料包括多层硅胶、氧化铝、弗罗里硅土等。目前,市场上可购买到的商品化多层硅胶柱成本较高,使用范围和可推广性受限。凝胶渗透色谱净化操作简单,但选择性不佳,而且溶剂消耗量过大。固相萃取柱可以在一定程度上大大减少有机溶剂消耗量,但是现有的商品化产品价格相对较高,一定程度上增加了土壤污染研究工作的经济成本。本课题组前期开发了基于氧化镁微球和氧化铝复合的固相萃取柱,可以实现对土壤中多氯萘的高选择性净化^[20]^。但是,氧化镁微球目前并没有实现商品化生产,在土壤污染调查研究中的应用受到一定程度的限制。因此,开发新型商品化吸附剂在多氯萘选择性净化方面的应用具有重要意义。X分子筛属于低硅铝比的沸石分子筛,因其易于合成,表面积大,亲水性好,且具有良好的离子交换和吸附性能,在干燥和分离方面得到了广泛应用。如X分子筛常用于吸附工业生产排放污水中的重金属离子和汽车排放的尾气,但在环境中新污染物净化方面鲜有报道^[21,22]^。我们发现13X分子筛可用于血清中新污染物与内源性物质的选择性去除^[23]^,如甘油酯、胆固醇等,但是尚不确定此方案是否适用于土壤提取液的净化。本研究旨在考察该固相萃取吸附剂在土壤中多氯萘选择性净化方面的适用性,探索其去除土壤中脂类等干扰物的效果。在此基础之上,建立了基于分子筛固相萃取的GC-MS/MS测定土壤中多氯萘的分析方法,并评估其方法适用性。

## 1 实验部分

### 1.1 仪器、试剂与材料

冷冻干燥仪(FREEZONE 4.5, 美国LABCONCO公司);旋转蒸发仪(R-205,瑞士Buchi公司);氮吹浓缩仪(DC-12,上海安谱公司);固相萃取仪(Visiprep DL,美国Supelco公司);气相色谱-三重四极杆质谱仪(TSQ Quantum XLS)和加速溶剂萃取仪(ASE-350)均购自美国Thermo Fisher公司;凝胶渗透色谱仪由P230高压稳流泵(大连依利特公司)、净化柱(600 mm×25 mm, SX-3 Bio-beads填料,美国Varian公司)和紫外检测器(BT3030,德国Biotronik公司)构成。

PCNs混合标准品(1 μg/mL CN-2、5、3、24、13、42、46、52、53、66、68、73、75);^13^C同位素标记的PCNs标准品(含1 μg/mL^13^C_12_-CN-42、27、52、67、73、75, 9.7 μg/mL^13^C_12_-CN-13, 10 μg/mL^13^C_12_-CN-2、6、13、64)(剑桥同位素实验室);丙酮、正己烷、二氯甲烷、壬烷(农残级,美国J. T. Baker公司);浓硫酸、氢氧化钾和无水硫酸钠(优级纯和分析纯及以上,天津市大茂化学试剂厂);硝酸银(分析纯,沈阳试剂二厂)。

13X分子筛固相萃取吸附剂(80~100目,阿拉丁试剂有限公司)、玻璃固相萃取柱和纤维筛板(3 mL)(博纳艾杰尔技术有限公司)、硅胶(100~200目,青岛恒泽硅胶制药有限公司);中性氧化铝(pH=7.5, MP Biomedicals公司)。

### 1.2 标准溶液的配制

1 μg/mL的多氯萘混合标准品用壬烷稀释成100 ng/mL的混合标准贮备液;10 μg/mL的同位素内标标准品溶液(^13^C_12_-CN-6、13、42、27、52、67、73、75)用壬烷配制成1 μg/mL的混合提取内标贮备液,用于条件优化实验;10 μg/mL的同位素内标标准品溶液(^13^C_12_-CN-2和^13^C_12_-CN-64)用壬烷配制成1 μg/mL的混合进样内标贮备液,用于条件优化实验。10 μg/mL的同位素内标标准品溶液(^13^C_12_-CN-2、6、13、42、27、52、67、65、73、75)用壬烷配制成1 μg/mL的混合提取内标贮备液,用于方法验证实验;10 μg/mL的同位素内标标准品溶液(^13^C_12_-CN-9和^13^C_12_-CN-64)用壬烷配制成1 μg/mL的混合进样内标贮备液,用于方法验证实验。上述贮备液均于-20 ℃冷藏保存。

### 1.3 制样、提取与浓缩

冷冻干燥:将采集的土壤样品放在搪瓷盘中混匀,除去枝棒、叶片、石子等异物,四分法取样后放入-20 ℃冰箱中至少冷冻24 h,然后置于冻干机中脱水;将干燥后的土壤样品研磨混匀后,密封保存在冰箱中。

提取与浓缩:称取10 g土壤,加入适量硅藻土和5 g铜粉研磨至流沙状,装入加速溶剂萃取池中以正己烷/二氯甲烷(1∶1, v/v)进行提取。萃取温度:100 ℃;萃取压力:2 MPa;静态萃取时间:10 min;循环次数:2次;溶剂体积:70%。旋转蒸发至干,用2 mL正己烷进行复溶。

### 1.4 净化、浓缩与定容

称取1000 mg 13X型分子筛装入3 mL玻璃小柱中,上下两端均用筛板封装;先用6 mL正己烷活化分子筛,然后将1.3节中所得溶液上样,继续用5 mL正己烷淋洗,弃去淋洗液;接着使用10 mL二氯甲烷/正己烷(2∶15, v/v)进行洗脱;所得洗脱液氮吹至干,加入进样内标(1 μg/mL混合进样内标贮备液10 μL),壬烷定容至200 μL,待气相色谱-串联质谱分析。

### 1.5 仪器分析

毛细管气相色谱柱为5%苯基-甲基聚硅氧烷(Rtx-5MS, 60 m×0.25 mm×0.25 μm),进样口温度为260 ℃,不分流进样,不分流时间为1 min。程序升温条件如下:初始温度80 ℃,保持1 min后以15 ℃/min的速率升温至160 ℃,继续以3 ℃/min的速率升温至265 ℃,再以5 ℃/min的速率升温至280 ℃,停留10 min。

质谱离子源为电子轰击源(EI),气相色谱和质谱接口的传输线温度为280 ℃,离子源温度240 ℃。扫描模式选择选择反应监测(SRM)模式,载气和碰撞气分别为高纯氦气和高纯氩气,定性、定量离子对和碰撞能量参见已发表文献^[1,24]^。

## 2 结果与讨论

### 2.1 13X分子筛对土壤提取液的净化效果

以13X分子筛为固相萃取吸附剂,正己烷为上样溶剂,20 mL二氯甲烷/正己烷(2∶15, v/v)为洗脱溶剂,考察该净化方法对土壤提取液的净化效果。从图1a和图1b中可以看出,棕黄色土壤提取液经过净化后,颜色透明,净化效果显著。将土壤提取液以及经过13X分子筛净化的等量土壤提取液分别采用凝胶渗透色谱进行检测。从图1c中可以看出,在有效的馏分收集时间内(22.2~41.5 min),土壤提取液中的绝大多数脂类可以被有效去除。不仅如此,我们将等量的土壤提取液分别采用凝胶渗透色谱、弗罗里硅土固相萃取、多层硅胶/氧化铝柱和13X分子筛固相萃取进行净化处理,比较其净化效果。从图2可以看出,经过凝胶渗透色谱和弗罗里硅土固相萃取后,所得提取液馏分浓缩后有明显固体附着在管壁上,净化效果不理想;而采用多层硅胶/氧化铝柱和13X分子筛净化后的土壤提取液相对比较干净。比较而言,13X分子筛的净化效果优于凝胶渗透色谱和弗罗里硅土柱。

**图1:**
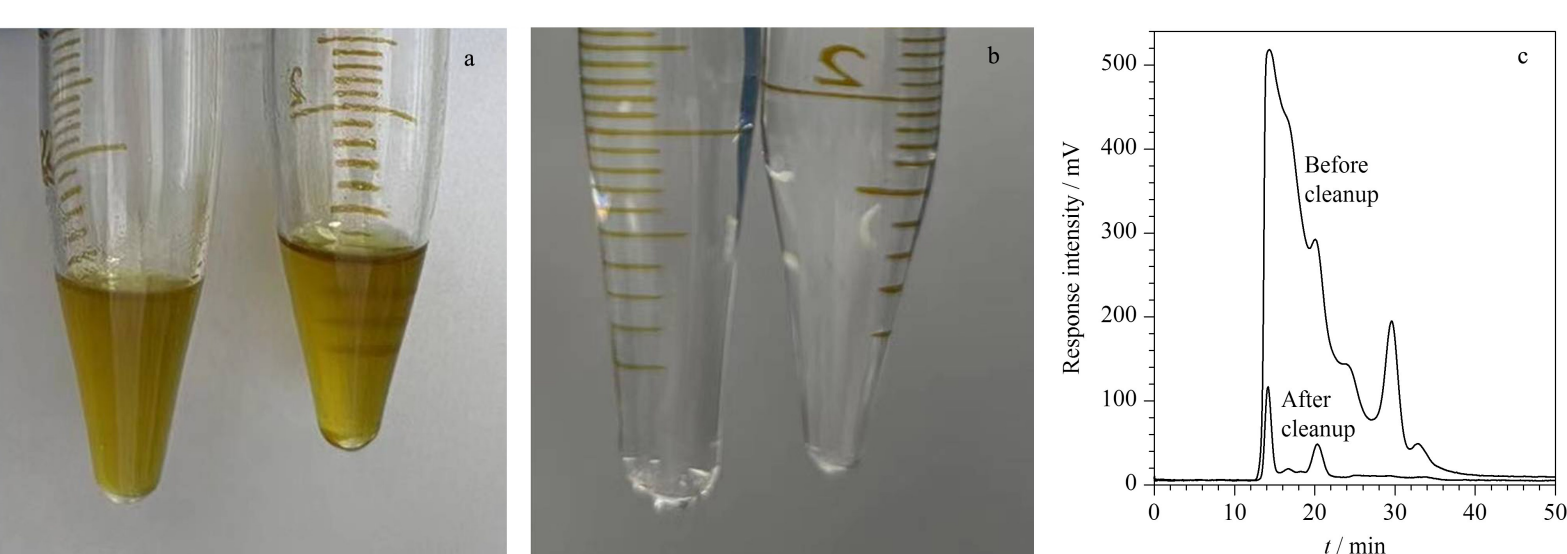
土壤提取液经13X分子筛净化(a)前、(b)后的颜色对比图及其(c)凝胶渗透色谱图

**图2:**
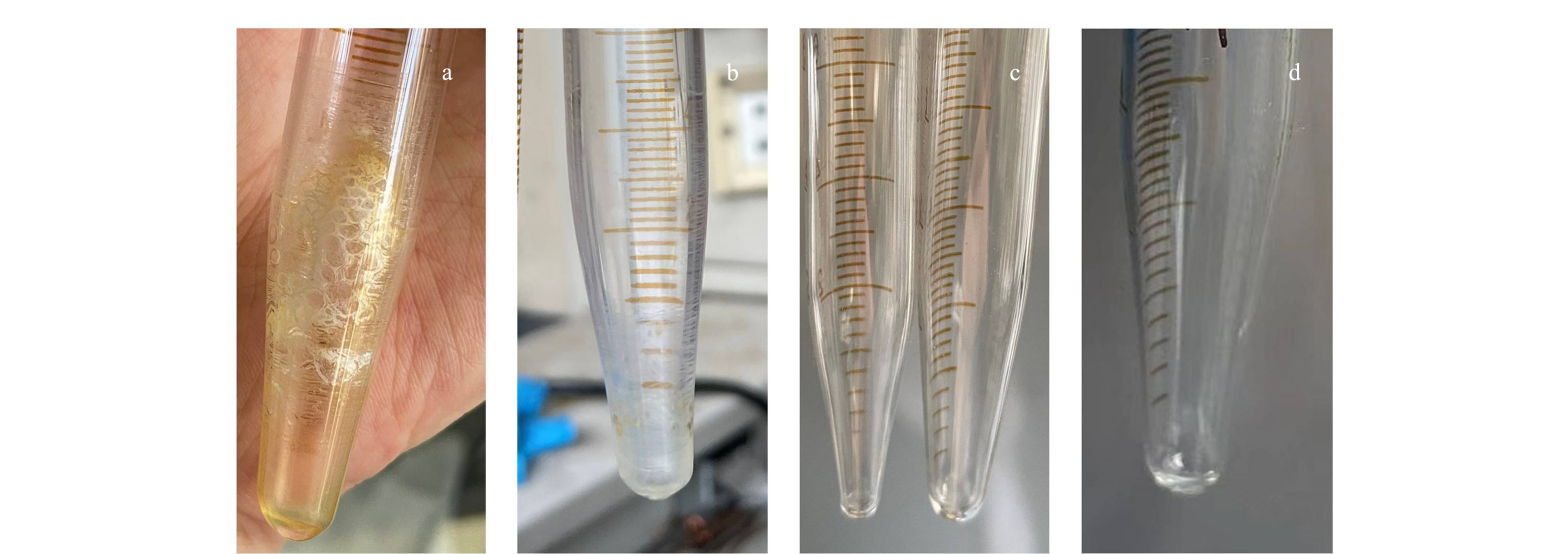
不同净化方法对土壤提取液的净化效果

随后,我们对加标的土壤提取液进行提取效率测定,结果见表1。从中可以看出:经过13X分子筛固相萃取净化后,PCNs提取内标的平均回收率范围为56.1%~88.0%,二氯萘回收率较低,三氯以上取代萘的回收率高于60%;经过多层硅胶/氧化铝柱净化后,PCNs提取内标的平均回收率范围为53.0%~117.0%,二氯萘回收率较低,四氯以上多氯萘基本可以实现完全回收(97.4%~117.6%)。两种净化方法相比,多层硅胶/氧化铝柱净化所得高氯萘的回收率较高,而13X分子筛净化后所得回收率虽然不如前者高,但是操作更加简单,有机溶剂用量更少(30 mL左右),环境更加友好,分析成本更低。

**表1:** 土壤提取液中多氯萘提取内标回收率(*n*=2)

Compound	Recoveries/%	
	Multilayer-silica and alumina column	13X molecular sieves
^13^C-PCN-6	53.0	56.1
^13^C-PCN-13	77.4	84.2
^13^C-PCN-42	76.8	65.6
^13^C-PCN-27	112.8	67.8
^13^C-PCN-52	117.0	76.4
^13^C-PCN-67	98.8	87.0
^13^C-PCN-73	102.6	88.0
^13^C-PCN-75	105.8	79.0

### 2.2 方法适用性验证

#### 2.2.1 标准曲线和仪器检出限

将PCNs混合标准溶液分别配制成质量浓度为2、5、10、20、50、100 ng/mL的系列标准溶液,运用已建立的GC-MS/MS方法对上述标准溶液进行3次重复测定。结果表明,在2~100ng/mL范围内,标准曲线的线性相关系数*R*^2^为0.9859~0.9995。选择系列浓度标准溶液中最低浓度的标准溶液进行5次以上重复测定,计算测定值的标准偏差,取标准偏差的3倍作为仪器检出限。仪器检出限用于估算方法检出限实验时的加标量。本研究中仪器检出限为0.2~1.2 pg。

#### 2.2.2 方法检出限

方法检出限(MDL)参考《环境监测分析方法标准制定技术指导》(HJ 168-2020)^[19]^中规定的方法进行评估。以空白土壤为基质,向其中添加1.0 ng PCNs标准物质,按照样品分析的全部步骤,重复7次试验;将各测定结果换算为样品中的含量(以ng/g 计),计算7次平行测定的标准偏差,取标准偏差的3.143倍作为方法检出限(保留一位有效数字)。除CN-5、24、46和68的方法检出限较高(0.2~0.6 ng/g)外,PCNs其他同族体的方法检出限为0.009~0.04 ng/g。

#### 2.2.3 精密度和准确度

取7份等量(10 g)的空白土壤,向其中6份土壤中平行加入一定量的目标物标准溶液(4、10、18 ng),按照提取、净化和分析全程序进行试验,平行测定6次。

如表2所示,CN-2、5和24加标回收率较低(3%~29%),且测定结果偏差大,即该方法不适用于这3种物质的准确定量。CN-66和CN-68在中、低浓度加标试验中,存在回收率较低(38%~48%)或高浓度加标试验中回收率过高(137%)的问题。其他同族体在低、中、高加标浓度下的平均回收率范围分别为70%~128%、71%~115%和61%~114%,测定结果的相对标准偏差分别为4.2%~23%、6.5%~31%和4.7%~22%。因此,从整个分析流程来看,该方法净化流程简单,可满足土壤中CN-3、13、42、46、52、53、73和75的准确定量。

**表2:** 土壤中多氯萘的平均加标回收率和相对标准偏差(*n*=6)

Analyte	0.4 ng/g				1.0 ng/g				1.8 ng/g				
	Found/(ng/g)	Recovery/%	RSD/%		Found/(ng/g)	Recovery/%	RSD/%		Found/(ng/g)	Recovery/%		RSD/%	
CN-2	0.01	3	93		0.05	5	54		0.07		4		78
CN-5	0.28	70	29		0.44	44	26		0.52		29		13
CN-3	0.28	70	7.0		0.71	71	10		1.10		61		11
CN-24	0.12	30	20		0.29^*^	29	28^*^		0.38		21		30
CN-13	0.33	83	5.1		0.86	86	8.5		1.47		82		9.6
CN-42	0.32	80	6.3		0.89	89	6.5		1.58		88		6.0
CN-46	0.51	128	23		0.92^*^	92	31^*^		2.02		112		22
CN-52	0.37	93	4.2		0.99	99	9.8		1.83		102		7.8
CN-53	0.28	70	5.2		0.79	79	8.3		1.27		71		11
CN-66	0.16	40	20		1.15	115	9.4		1.96		109		4.7
CN-68	0.19	48	20		0.38^*^	38	17^*^		2.47		137		17
CN-73	0.34	85	7.4		0.85	85	10		1.59		88		7.2
CN-75	0.43	108	8.2		1.11	111	11		2.06		114		11

* Statistical data after dropping outlier data.

### 2.3 分析方法比较

方法检出限在一定程度上受样品前处理流程、取样量、定容体积和仪器检出限等影响,简单地进行数值比较没有实际意义。为此,表3列出了用于土壤中多氯萘测定的GC-MS/MS分析方法细节。从中可以看出,采用3倍信噪比方法往往可以获得较低的方法检出限,而通过增加取样量,缩小定容体积,也可以在一定程度上降低方法检出限。为了提高对比结果的有效性,本研究分别采用了13X分子筛固相萃取和层析柱(多层硅胶柱和氧化铝柱)对土壤样品进行前处理,采用相同的仪器和数据处理方法进行数据分析。研究发现,在层析柱法处理后的土壤样品中低氯萘回收率较低,测定结果偏差较大,所以表格中仅统计了氯原子数目为4以上的多氯萘的回收率。与层析柱法相比,经过13X分子筛固相萃取后,CN-5、24、46、68的方法检出限较高(0.2~0.6 ng/g),其余多氯萘的方法检出限与层析柱法相当。

**表3:** GC-MS/MS测定土壤中多氯萘的分析方法比较

Extraction	Cleanup	Cl number	Sample mass/g	Constant volume/μL	Recovery/%	MDLs			Ref.
						Calculation	Value/(pg/g)		
Soxhlet	CC	3-8	10	200	63-146	3 S/N	0.03-	0.52	[1]
ASE	CC	1-8	10	200	6.5-116	3 S/N	0.28-	17.2	[7]
ASE	CC	1-8	10	200	6.1-125	3.143 S	0.28-	17.2	[17]
PLE	CC	1-8	15	100	55.6-104.5 (tetra- to octa-CNs)	3.143 S	0.26-	1.6	[18]
ASE	CC	1-8	10	40	45.2-87.9	3.143 S	0.04-	1.92	[25]
ASE	SPE	1-8	10	200	65-137 (tetra- to octa-CNs)	3.143 S	20-	600	this study
ASE	CC	1-8	10	200	59.5-115.5 (tetra- to octa-CNs)	3.143 S	21-	83	this study

ASE: accelerated solvent extraction; PLE: pressure liquid extraction; CC: column chromatography; SPE: solid-phase extraction; CNs: chlorinated naphthalenes; MDLs: method detection limits; *S*: standard deviation.

## 3 结论

13X分子筛作为一种价格相对低廉的吸附材料,可以较好地应用于土壤提取液中多氯萘的选择性净化。通过13X分子筛固相萃取,在正己烷上样体系下,使用10 mL二氯甲烷/正己烷(2∶15, v/v)可以洗脱PCNs,实现目标物与脂类等大分子干扰物的选择性分离,达到良好的净化效果。本研究建立的基于13X分子筛固相萃取法的多氯萘分析方法,操作简单,环境更加友好,可满足土壤中CN-3、13、42、46、52、53、73和75的准确定量,有望在土壤提取液中新污染物净化分离方面发挥重要作用。
